# Postoperative urinary stone analysis—influence of the hydrogel retrieval method on stone analysis

**DOI:** 10.1007/s00345-025-05831-x

**Published:** 2025-08-01

**Authors:** C. Jacobs, F. I. Winterhagen, M. Ritter, P. Lossin, I. Grunwald, J. Stein, S. Latz

**Affiliations:** 1 Department of Urology, University Hospital Bonn, Venusberg Campus 1, 53127 Bonn, Germany; 2 Urinary Stone Analysis Center, Urologie Bonn-Rhein-Sieg, Bonn, Germany; 3 Industrial and Environmental Biology, Hochschule Bremen-City University of Applied Sciences, Neustadtswall 30, 28199 Bremen, Germany

**Keywords:** Urolithiasis, Metaphylaxis, Stone analysis, Hydrogel

## Abstract

**Purpose:**

Stone analysis is crucial in patients with urolithiasis in order to initiate specific metaphylaxis in patients with high risk of recurrence. Hydrogel (mediNik^®^, Farco) was developed in order to improve stone-free rates. The aim of this study was to investigate whether the use of hydrogel influences the stone analysis.

**Methods:**

This randomized prospective blinded study was based on a total of 78 urinary stone analyses, the composition of which was measured before and after treatment with hydrogel. Samples were washed according to the manufacturer’s instructions (group 1), with EDTA only (group 2) or not washed after treatment with hydrogel (group 3). The samples were analyzed using Fourier transform infrared spectroscopy (ATR technique).

**Results:**

In group 1 (100%, 63) and group 2 (100%, 5), the same urinary stone findings were recorded before and after application of the hydrogel. In group 3, the composition of none of the stones could be determined due to additional bands.

**Discussion:**

Postoperative urinary stone analysis using IR spectroscopy is not impaired by the hydrogel if the hydrogel is washed out as recommended by the manufacturer. After regular washing, the stone analysis remains the same in 100% of cases with and without the use of hydrogel.

## Introduction

### Background

Urolithiasis represents one of the most frequent urological diagnoses worldwide. The prevalence increased significantly in the industrial nations in the last decades [1–4]. Correspondingly, the amount of surgery and interventions due to urinary stones or related complications rose tremendously [5]. It is thereby not only an individual issue due to repeated interventions, possible complications such as sepsis, ureteral stenosis, bleeding and even terminal renal insufficiency, but also an enorcmous social and economic burden due to treatment costs and loss of working hours.

The recurrence rate without targeted therapy ranges from 50 to 80%. In high risk stone formers international guidelines agree to recommend a specific stone metaphylaxis, which is capable to reduce recurrence risk to 10–15% [6–8]. Stone analysis is crucial to identify risk factors (e.g. uric acid, struvite) and determine the strategies for specific metaphylaxis. Thus, stone analysis is recommended in current guidelines e.g. in the European EAU guideline on urolithiasis for every first stone event and in case of recurrence under metaphylaxis, rapid stone recurrence, and stone recurrence after a long stone-free period.

In this context, residual stones after stone treatment gained the focus of the urological society recently. Several lines of evidence proofed, that residual fragments are an important risk factor for stone recurrence [9]. Reported stone-free rates for e.g. ureteroscopic procedures range between 38.3% and 75.0% [10–12]. Small fragments, which are difficult or impossible to remove via basket, are often left behind in the assumption that they will pass spontaneously after ureterorenoscopy. However, a study from Kang et al. showed that only 40% of patients with residual fragments < 1 mm and only 25% of patients with residual fragments < 3 mm spontaneously passaged the fragments within 2 years [13]. Small fragments can recombine and lead to recurrent symptoms or serve as a nidus for the formation of larger stones [14].

Keller et al. recently defined dust as particles ≤ 250 µm that can be aspirated through a 3.6 Fr. working channel of a modern ureterorenoscope and have a reasonable chance of passing spontaneously after the procedure [15]. Nevertheless, the intraoperative identification of fragments, that do not fulfill the criteria of dust, is often difficult due to poor visibility and phenomena such as the snow globe effect [16]. Lithotripsy of very small fragments into dust is also challenging due to dislocation of these fragments by irrigation flow and laser impulse. Therefore, the development of technologies for achieving a stone-free status has been driven forward in recent years.

In the past, approaches have been explored in this context that have not become established in clinical practice. For instance, autologous blood was applied to the stone fragments. After clotting, the fragments could be extracted as they were stuck in the blood clot. It was first described during open pyelolithotomy [17]. However, the use was restricted in the middle 1980s. The use of autologous blood regained attention 2005 [18]. However, it has not become established in routine clinical practice as it is associated with the risk of colic if not all residual blood clots are removed from the kidney, prolonged procedure time and impaired visibility.

A modern method for stone retrieval involves the use of hydrogels [19, 20]. These substances bind to the small stone material, gels, and facilitate removal. It offers the advantage of effectively binding small stone fragments and facilitating their removal without the need for additional materials. In contrast to the previously mentioned blood-clot method, residual hydrogel in the kidney dissolves with urine, gelation takes less than 3 min, and visibility is good due to coloring. However, it is not yet known whether the hydrogels change the results of stone analysis.

### Hydrogel

In this study, we used a biocompatible hydrogel consisting mainly of plant polysaccharides called mediNiK^®^ (FARCO-PHARMA GmbH). The basis of the material used in this study consists of the biopolymer alginate. This consists of repeating units of the two sugar molecules β-D-mannuronate (M) and α-L-guluronate (G), which are linked together via a (1,4)-glycosidic bond. The number of individual monomers can range from a few hundred to several thousand units, with individual regions usually consisting of blocks made up of consecutive M residues (MM…), consecutive G residues (GG…), and alternating G and M residues (MGMGMG…) [24]. These long chains (GG… blocks) can be physically/ ionically cross-linked by the addition of divalent metal ions, such as calcium (Ca^2+^), and thus gelled, whereby the respective composition of the G and M blocks plays an important role in the physical properties of the hydrogel formed. The individual blocks of the α-L-guluronate, for example, are largely responsible for the subsequent strength of the hydrogel formed [25]. The special type of gel formation through a purely physical process (no chemical bonds are formed within this process within the gel) of, for example, calcium-mediated cross-linking makes it possible to dissolve the formed hydrogel again by removing the divalent metal ion, i.e., to return it to its original state. Citrate and ethylenediaminetetraacetic acid (EDTA) or hexametaphosphate [21] are a group of substances known as chelators, some of which are also used in clinical applications. The biocompatible hydrogel described in this publication for the removal of residual stones after ureterorenoscopy (URS) is CE certified and has already been described in various publications [19, 22, 23] and in a clinical study (DRKS-ID DRKS00030532).

## Methods

The study underwent review by the Ethics Committee of the University of Bonn, which concluded that ethical vote is not required (361/23-EP).

This is a prospective, blinded, randomized study performed at the Urinary stone Analysis Center in Bonn, Germany. The study was based on a total of 78 urinary stone analyses, the composition of which was measured in the laboratory before and after treatment with hydrogel (mediNiK^®^, FARCO-PHARMA GmbH).

Prospective, randomized recruitment was carried out until all relevant urinary stone types (including common mixed stones) were repeatedly present and included whewellite, weddellite, carbonate apatite, brushite, struvite, uric acid, uric acid dihydrate, ammonium urate, cystine.

The urinary stones received in the urinary stone laboratory and used for this study generally range in size from 1 to 15 mm. However, in clinical practice, hydrogel is used to remove very small fragments from the renal pelvicalyceal system, which are usually smaller than 2 mm. Therefore, the size of the stones used in this study was adjusted; prior to contact with the hydrogel, they measured between 1 and 2 mm.

After initial measurement of the stone composition, the urinary calculi were placed in the hydrogel for 3–4 h and then subjected to various washes. 63 samples (group 1) were washed in 5–10 mL of a 0.5 molar EDTA solution according to the manufacturer’s instructions until the hydrogel clot was completely dissolved. This takes a few minutes. The samples are then washed briefly in water. A further 5 samples were washed with EDTA only (group 2), while 10 samples (group 3) were not washed at all after the contact time with the hydrogel. Afterwards samples underwent a new measurement and blinded evaluation. Washing procedure is ideally performed by the user in the operating room. However, urinary stone laboratories should be aware of the increasing use of medinik. If gel residues are present in the laboratory, the laboratory can and must recognize this and be aware of the washing process. Furthermore, the laboratory must be aware of the artifact bands in order to be able to wash again in case of doubt.

**Fig. 1:**
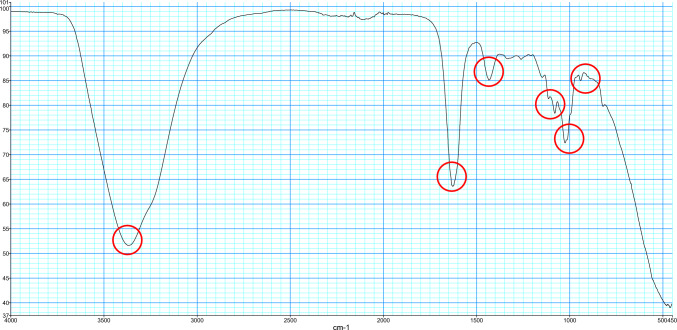
mediNiK hydrogel analyzed using Fourier-transform infrared spectroscopy (FTIR) in ATR technique, results are shown in analysis spectra. Typical for mediNiK hydrogel are the bands at 3369, 1627, 1434, 1079, 1024 and 821 cm^−1^ (indicated with red circles)

The samples were analyzed using Fourier-transform infrared spectroscopy (FTIR) in attenuated total reflection (ATR) technique (FTIR-ATR), results are shown in analysis spectra (Fig. 1). By knowing the bands of the different stone materials, the stone composition can be determined. In case of mixed stones, the individual components were semiquantitatively indicated in 5% increments. This allowed a detailed assessment of the varying composition within mixed stones and was personally conducted by the laboratory management after IT-assisted pre-evaluation. This ensured a meticulous and standardized approach to the analysis of each urinary stone sample. The two spectra were correlated using the “Spectrum.Ink” measurement software (PerkinElmer “Spectrum Two” device, software 10.7.2, PerkinElmer Inc., USA).

Data analysis was performed using IBM SPSS Statistics v27. Descriptive statistics were calculated for categorical variables, including frequencies and proportions, while continuously coded variables were described with means and standard deviations. An unpaired, two-tailed t-test was used to test statistical significance (p < 0.05).

## Results

### Analysis spectra

**Fig. 2:**
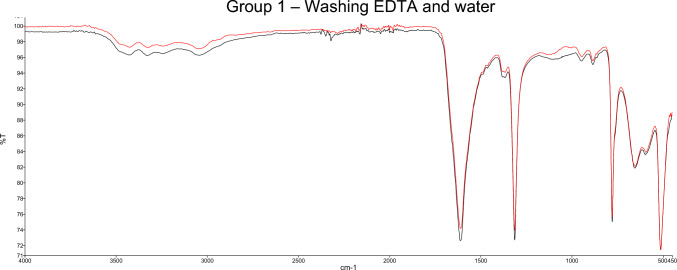
Example for analysis spectra using Fourier-transform infrared spectroscopy (FTIR) before (black) and after (red) application of the hydrogel. Washing with EDTA and water was performed (group 1). The typical bands of the hydrogel cannot be seen. Correlation of the two curves: 0.9966. Stone composition: 90% whewellite, 10% weddellite

The stone composition was measured before and after treatment with hydrogel and the analysis spectra were compared. In group 1, where the hydrogel was washed out with EDTA and water according to the recommended procedure both curves correlated on average by 0.983 ± 0.0156 standard deviation. No artifact bands were detected. Results of the FTIR-ATR spectra are shown in Fig. 2.

In group 2 where the samples were washed with EDTA only both curves correlated by 0.819 ± 0.258 standard deviation. In 4 of 5 cases artificial bands could be seen which did not represent the known bands of the hydrogel but might be due to EDTA rest.

**Fig. 3:**
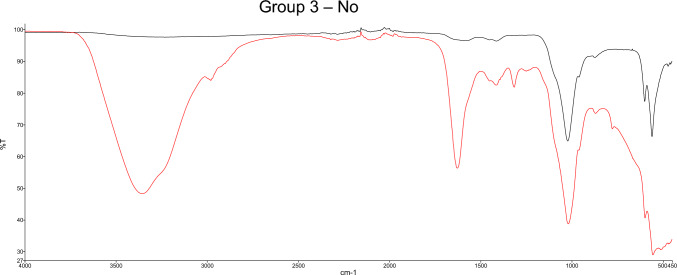
Example for Analysis spectra using Fourier-transform infrared spectroscopy (FTIR) before (black) and after (red) application of the hydrogel. No washing was performed. The typical bands of the hydrogel can be seen at 3369, 1627 and 1434 cm^−1^. Correlation of the two curves: 0.4956. Stone composition: 100% carbonate apatite

In group 3, where the samples were not washed, the analysis spectra show overlaps with the known mediNiK^®^ bands (3369, 1627, 1434, 1079, 1024 and 821 cm^−1^) (see Figs. 1 and 3).

Comparing the groups with each other, group 1 differed significantly (p < 0.001) from groups 2 and 3. Group 2 and 3 did not differ (p = 0.997).

### Composition of urinary stones

The analysis spectra allow to determine the composition of the stones. After interpretation of the spectra, the same stone composition was found both before and after the application of hydrogel for samples from group 1 (100%, 63) and group 2 (100%, 5). In group 3, the composition of none of the stones could be validly determined due to the artificing bands caused by the hydrogel.

## Discussion

Residual stones after endoscopic stone interventions are common and contribute to significant urinary stone recurrences [9, 26]. Various approaches have recently been developed to address this issue.

In this context suction-based techniques have been developed and some have already been established in clinical practice [27–29]. Flexible and Navigable Suction (FANS) in particular has been the subject of recent studies. By using ureteral access sheaths, which are flexible at the tip, suction can be navigated to the stones in the individual calyces. This enables the suction of dust during laser lithotripsy. In addition, larger fragments can be aspirated by retracting the URS via the access shafts without the additional use of a bascet or forceps. Another approach involves the direct suction of stone particles through the working channel of the URS, a technique commonly referred to as Direct In-Scope-Suction (DISS) [30, 31]. However, the wide range of published stone-free rates with suction-based approaches, varying from 46.4 to 80%, indicates that optimal, reproducible outcomes have not yet been achieved [28–30].

Concurrently, hydrogel (mediNiK^®^, FARCO-PHARMA GmbH) represents a novel non-suction-based approach that has been introduced and launched on the market, aiming to improve stone-free rates.

To evaluate the safety, tolerability and performance data of the hydrogel method, an open, randomized, multicentric study [32, 33] was conducted, this in comparison with the standard of care (SOC) for the removal of kidney stones.

This study found no safety concerns with the hydrogel method and its results support the statements of the Clinical Evaluation regarding the safety of the product. No difference in safety was found between the hydrogel method and the standard URS procedure.

The primary analysis showed that in the hydrogel group (n = 23) a total of 1716 stones, or 74.61 stones per subject, smaller than 1 mm were removed. In the URS group (n = 17), a total of 209 stones, or 12.29 stones per subject, smaller than 1 mm were removed. This means that, on average, a clear tendency of higher number stones smaller than 1 mm were removed with the hydrogel group than in the URS group. These results can be explained by the fact that stone fragments of small size can hardly or not at all be grasped with the help of the conventionally used graspers. The hydrogel method enables the removal of these small fragments.

However, data on the stone free rates (SFR) determined by postoperative CT-scan or ultrasound are not available.

Urinary stone analysis plays a crucial role in determining the cause of urinary stones and initiating appropriate prophylactic therapies, so it is necessary that the hydrogel used does not alter the stone composition result.

After washing off the hydrogel with EDTA and water, the analysis spectra before and after using the hydrogel showed a high correlation (0.983 ± 0.0156 standard deviation), the stone analysis remained the same in 100% of the cases with and without using the hydrogel.

When the hydrogel is not washed with EDTA and water, artificial bands are formed by the remaining hydrogel. This can make it difficult or impossible to analyze the stone composition: The spectra changed before using the hydrogel and after washing with EDTA only (0.819 ± 0.258) or without washing (0.819 ± 0.174 standard deviation).

Still, after washing with EDTA only, the results for the determination of stone composition were possible as before application of the hydrogel but was made more difficult by artifacts.

Due to the additional artifact bands, the analysis of group 3 showed analytical spectra from which the stone composition could not be determined.

There is no evidence that the material of which the stones are composed itself influences the analysis; however, it seems crucial to ensure that all gel residues are removed.

## Conclusion

Postoperative urinary stone analysis using FT infrared spectroscopy in ATR technique is not affected by using the hydrogel mediNiK® if the hydrogel is washed out with EDTA and water as recommended by the manufacturer. After proper washing, there is no significant difference in the analysis spectra. Even with a single wash with EDTA, the final analysis results representing the stone composition are identical. Without washing, it is not possible to determine the stone composition using Fourier transform infrared spectroscopy in ATR technique. This is most likely due to overlapping or additional peaks in the FTIR-ATR spectra obtained. Washing with EDTA and water should be performed; however, urinary stone laboratories should be aware of possible artifact bands and know how to remove e.g. gels or other drug residues from previous treatments or at least be informed before sending the stone for analysis.

## Data Availability

No datasets were generated or analysed during the current study.
